# MicroTools enables automated quantification of capillary density and red blood cell velocity in handheld vital microscopy

**DOI:** 10.1038/s42003-019-0473-8

**Published:** 2019-06-19

**Authors:** Matthias Peter Hilty, Philippe Guerci, Yasin Ince, Fevzi Toraman, Can Ince

**Affiliations:** 1 Department of Intensive Care, Erasmus MC, University Medical Center, Rotterdam, 3015GD The Netherlands; 2 Department of Anesthesiology and Reanimation, Acıbadem Mehmet Ali Aydınlar University School of Medicine, Istanbul, 34752 Turkey

**Keywords:** Cardiovascular biology, Image processing, Computational models, Translational research

## Abstract

Direct assessment of capillary perfusion has been prioritized in hemodynamic management of critically ill patients in addition to optimizing blood flow on the global scale. Sublingual handheld vital microscopy has enabled online acquisition of moving image sequences of the microcirculation, including the flow of individual red blood cells in the capillary network. However, due to inherent content complexity, manual image sequence analysis remained gold standard, introducing inter-observer variability and precluding real-time image analysis for clinical therapy guidance. Here we introduce an advanced computer vision algorithm for instantaneous analysis and quantification of morphometric and kinetic information related to capillary blood flow in the sublingual microcirculation. We evaluated this technique in a porcine model of septic shock and resuscitation and cardiac surgery patients. This development is of high clinical relevance because it enables implementation of point-of-care goal-directed resuscitation procedures based on correction of microcirculatory perfusion in critically ill and perioperative patients.

## Abstract (web summary)

Matthias Hilty et al. develop MicroTools, a computer vision algorithm that analyzes image sequences obtained from handheld vital microscopy. MicroTools can provide morphometric and kinetic information about the sublingual microcirculation in real-time, making this tool highly relevant in clinical settings.

## Introduction

In critically ill and perioperative patients, the main objective of resuscitation is to recruit the microcirculation^1,2^. The surrogates that are in clinical use are arterial blood pressure and peripheral perfusion. Techniques for measuring the microcirculation have improved substantially and have evolved from methods that are limited in scope, such as velocity-based laser Doppler^3^ and near-infrared spectroscopy^4^, to handheld vital microscopy (HVM). With imaging technology having progressed from orthogonal polarization spectral to sidestream dark field and incident dark field imaging, HVM can directly visualize the flow of red blood cells^5,6^, thereby demonstrating that alterations in microcirculatory function in states of shock and during surgery affect the outcome^7,8^. Application on the sublingual microcirculation has proven especially relevant in a clinical setting^9^. While goal-directed therapy on arterial blood pressure has failed to demonstrate improved survival^10–12^, various forms of alterations of the microcirculation and functional parameters^13^ have been identified via manual analysis of HVM image sequences and provide insight into possible treatment targets^14^. However, to target the microcirculation during resuscitation, automated real-time analysis capability of HVM image sequences is required, as was recently stated in an international consensus paper published by the European society of Intensive Care Medicine^9^. Automated image sequence analysis has proven difficult to develop mainly due to the inherent complexity of the content. Hence, previous attempts at creating algorithms for measuring the total vessel density (TVD) and functional capillary density (FCD) as measures of the microcirculatory diffusion capacity have not been successfully validated. Thus, manual analysis using the AVA 3.2 software package (Advanced Vascular Analysis, Academic Medical Center, University of Amsterdam)^15^, which is a tool that primarily facilitates manual analysis via a process that requires approximately 20 min per image sequence^16^, has remained the gold standard for vessel recognition. The assessment of red blood cell displacement as a measure of the microcirculatory convection capacity using current tools represents an even bigger challenge. Although measurement of the absolute red blood cell velocity (RBCv) of selected capillaries has been realized using manual space–time diagram analysis^17^, applying this manual method to all capillaries rendered in an HVM image sequence for accurate and unbiased representation of RBCv within the field of view is not feasible. Thus, subjective and qualitative surrogate parameters for RBCv have been developed, such as the per-quadrant or per-capillary microcirculatory flow index (MFI)^18,19^. It has been demonstrated that a per-capillary application of MFI better represents RBCv than a per-quadrant application^20^ and per-capillary assessment of MFI is used to determine FCD as a measure of perfused capillary density and the proportion of perfused vessels (PPV). However, information in HVM image sequences that more accurately reflects the convection component of microcirculatory function is entirely inaccessible using current tools, such as the absolute RBCv within the field of view or even more the distribution of the absolute RBCv across individual vessels within the field of view.

Since the early phase of microcirculatory research, two main factors have opened the door to employing complex algorithms on large datasets such as HVM image sequences: an increase in computer processing power by seven orders of magnitude within 30 years and the development of advanced computer vision algorithms, in conjunction with ongoing industrial digitalization, that are available for further development by the scientific community due to their open-source nature^21^. Building on these achievements, a novel advanced computer vision algorithm and a fully automated software tool called MicroTools were developed to analyze HVM image sequences without human intervention and objectively extract the parameters given in Table 1. Our hypotheses are that (I) automated recognition of vessels and, thus, measurement of TVD in HVM image sequences using advanced computer vision techniques is equivalent to manual analysis; (II) the perfusion state of a single capillary, as defined in the current consensus by the subjective, qualitative MFI score, can be represented by the cutoff value defining the presence of microcirculatory pathology of the absolute RBCv, as measured by a space–time diagram within that capillary; and (III) automated space–time diagram analysis is equivalent to manual analysis, thereby enabling automated algorithm-based measurement of FCD and PPV and objective measurement of RBCv in HVM image sequences. Our objective in this paper is to describe the algorithm and test our hypotheses by validating the algorithm against manual analysis using the AVA 3.2 software on sublingual HVM image sequences in a porcine model of septic shock and on cardiac surgery patients following the initiation of cardiopulmonary bypass.

**Table 1:** Objective parameters of the microcirculation computed from vessel detection and space–time diagram analysis using the proposed advanced computer vision algorithm

Symbol	Unit	Description	Physiological context
*Per field of view*			
TVD, total vessel density for capillaries	mm mm^−2^	Sum of the length of all capillaries containing red blood cells, divided by field of view	Measure of microcirculatory diffusion capacity
PPV, proportion of perfused vessels for capillaries	1	Weighted mean (by vessel length) of the categorical per-vessel “non-perfused” property, which describes a per-space-time-diagram-ridge velocity frequency histogram area under the curve proportion threshold transgression, after artifact elimination	Basis for FCD calculation
FCD, functional capillary density	mm mm^−2^	Sum of the length of all capillaries containing moving red blood cells, divided by field of view	Similar to PVD, with the advantage that it does not suffer from inconsistent definitions based on subjective categorical evaluation of capillary flow velocity
RBCv, mean capillary velocity	µm s^−1^	Weighted mean (by vessel length) of the absolute red blood cell velocity in all capillaries within the field of view	Absolute blood flow velocity as a measure of microcirculatory convection capacity
*Per vessel*			
Length	µm	Vessel length along the centerline	
Mean diameter	µm	Mean of all diameter measurements perpendicular to the centerline	
Mean RBCv	µm s^−1^	Mean of all RBCv measurements in non-discarded RBC paths within one vessel	
*v*_max_	µm s^−1^	Maximum detectable RBCv given the length of the vessel and frame rate of the image sequence	
Vessel type	Categorical	{capillary, venule}	
Perfusion type	Categorical	{perfused, non-perfused}	
*Per RBC path*			
RBCv	µm s^−1^	First derivative by time for an individual RBC path	
RBC path type	categorical	{no flow/low flow, normal flow, artifact}	
Curvature index	1	Proportion of length of straight line between start and endpoint of RBC path, and actual RBC path length	

## Results

### Vessel recognition workflow

**Fig. 1:**
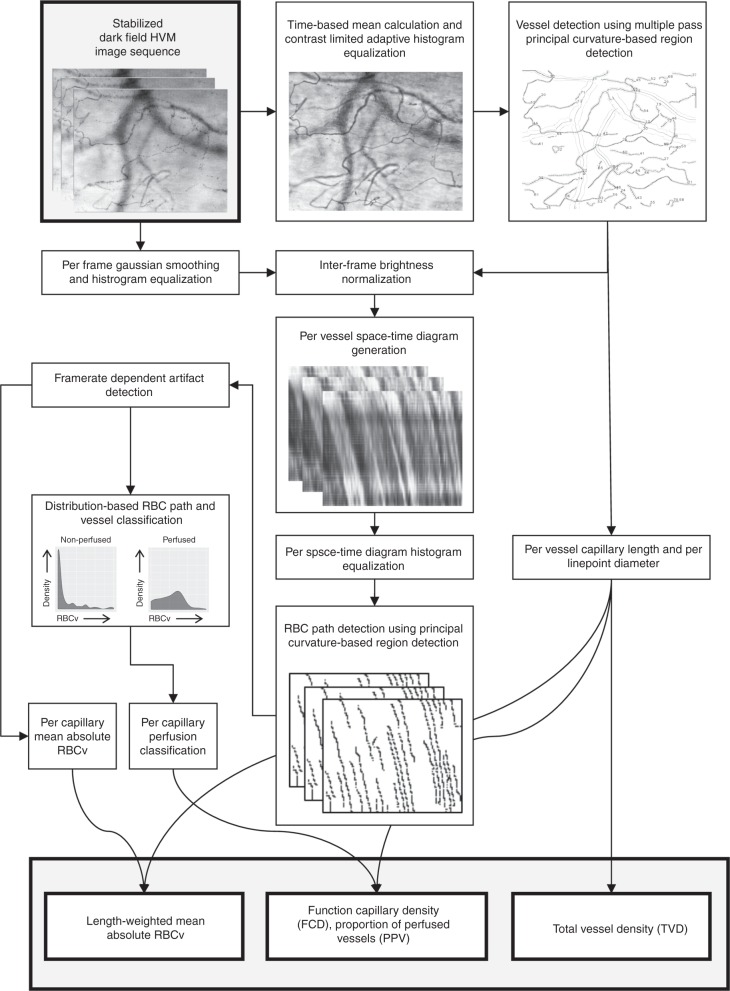
Schematic representation of the proposed advanced computer vision algorithm. The flowchart is illustrated with representative examples from the validation dataset

**Fig. 2:**
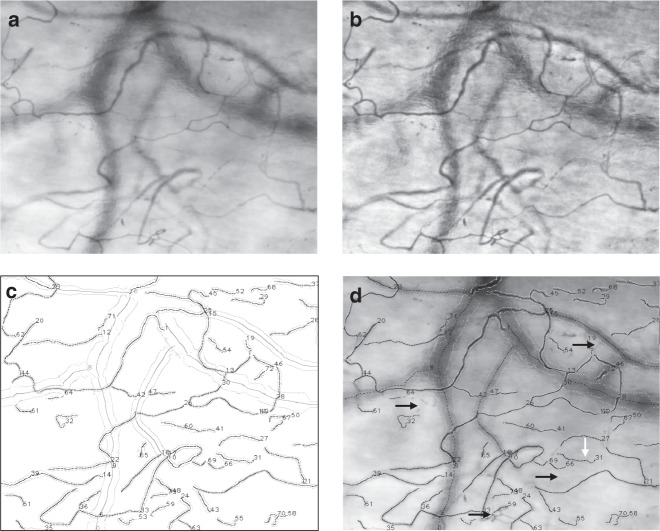
Representative example of a mean image (**a**) and context-aware contrasted mean image (**b**) generated from a stabilized HVM image sequence. Algorithmically recognized vessel structures are visualized in **c**, while superimposition of the detected vessel structures onto the mean image demonstrate short segments of false-negative (**d**, black arrows) and false-positive (**d**, white arrows) vessel recognition. Recognized capillary and venular centerlines are marked as black and dark gray lines (**c**) or dashed lines (**d**), outer vessel delineations are marked as light gray lines

HVM imaging directly visualizes hemoglobin that is contained in red blood cells, as opposed to anatomical structures of the tissue, thereby defining the functional component of the microcirculation in terms of oxygen carrying capacity. Thus, the boundaries of microvascular structures within an HVM image sequence are defined by the spatial arrangement and direction of movement of red blood cells, and structures not currently perfused by red blood cells may only be visualized using recruitment maneuvers as described elsewhere^22^. These structures, which may be further categorized into arterioles, capillaries, and venules in subsequent steps, are referred to as “vessels” in the current manuscript. To optimize vessel recognition, frame averaging is utilized as a technique to reduce the impact of plasma gaps between consecutive red blood cells on vessel recognition in HVM image sequences^15^. The MicroTools software package computes a mean image from the stabilized HVM image sequence based on per-pixel grayscale values (Figs. 1 and 2a). Contrast-limited adaptive histogram equalization is applied to the mean image to effectively reduce the influence of uneven lighting or background structures, thereby increasing the signal-to-noise ratio in vessel recognition (Fig. 2b). A combination of first- and second-derivative Gaussian kernel convolutions with the contrast-enhanced time-based mean image and an orientation-based linking algorithm then yields the centerlines and diameters of the recognized vessels (Fig. 2c). The mean vessel diameter is used for classification into capillaries (≤20_;_μm) and venules according to the consensus requirements^9^. TVD is calculated in concordance with current consensus^9^ as the sum of the lengths of all detected capillaries divided by the field of view. In the literature, the term TVD has been inconsistently used to refer to the density of vessels of varying diameters; in the current study, it explicitly refers to the capillary density as reflecting that part of the microcirculation as being primarily responsible for oxygen transport to the tissues.

### Absolute RBCv and capillary perfusion

**Fig. 3:**
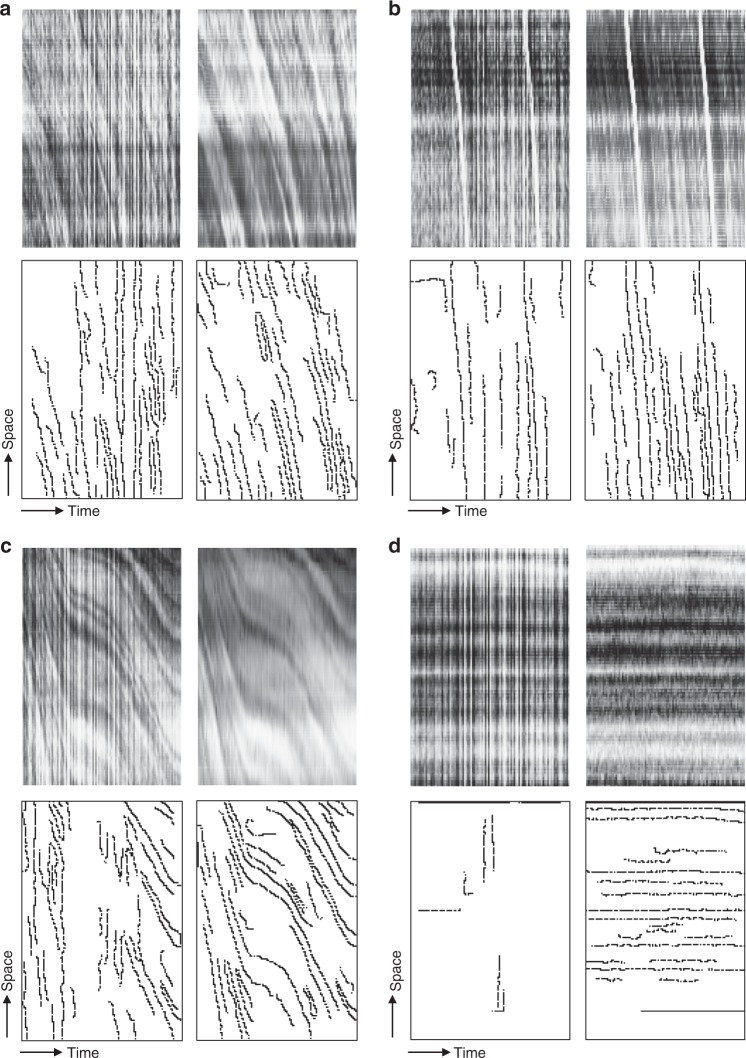
Representative examples of algorithmically generated space–time diagrams and corresponding detection of individual RBC paths before and after frame-by-frame image enhancement. Top row (**a**–**d**) represent algorithmically generated space–time diagrams; in the bottom row (**a**–**d**) individual RBC path detection is shown. Top left images (**a**–**d**) represent space–time diagrams produced without further image processing and demonstrate artifacts introduced by inter-frame variability in brightness (“flickering”) that translate into individual RBC path detection artifacts (bottom row, **a**–**d**). Results of frame-by-frame image enhancement are shown on top right (**a**–**d**), demonstrating marked reduction in individual RBC path detection artifacts (bottom row, **a**–**d**). Representative examples are given for capillaries with normal flow (individual vessel MFI = 3, **a**); “hyperdynamic” flow (**b**), demonstrating reliable detection of RBC velocity even when approaching *v*_max_; intermittent flow (individual vessel MFI = 1, **c**); and no flow (“barcode sign”, individual vessel MFI = 0, **d**)

In the proposed algorithm, velocity estimation of red blood cell movement along a vessel in the present algorithm is based on space–time diagrams^23,24^, which are two-dimensional representations of grayscale values along a straightened vessel centerline that form the vertical axis and a horizontal time axis (Figs. 1 and 3). Individual red blood cell paths are identified within space–time diagrams; this is analogous to vessel recognition in the mean image. After artifact elimination, the red blood cell path velocity is represented by the mean slope. The vessel RBCv is equal to the mean velocity of the red blood cell paths contained therein and a vessel is considered perfused based on a per-vessel density distribution of red blood cell path velocities. RBCv over the entire field of view is computed as a weighted mean of the capillary RBCv by capillary length to avoid the introduction of bias by three-dimensional volume-to-focal plane translation, such as in the recording of an HVM image sequence that depicts a capillary network. Depending on the position of the focal plane, a capillary with high RBCv could be represented as several short segments, whereas a capillary of equal length with low RBCv could be represented as a single long segment, thereby over-representing the former capillary in the velocity distribution within the field of view. Based on the capillary perfusion classification, FCD is calculated as the sum of the lengths of all perfused capillaries divided by the field of view. Consequently, PPV is calculated as the length-weighted mean of the categorical per-vessel perfusion states and represents the quotient of FCD and TVD.

### Validation in a standardized model of septic shock

The proposed algorithm was validated against manual analysis using the AVA 3.2 software on HVM image sequences that were obtained from sublingual microcirculation observations in a porcine model of septic shock and resuscitation^25^. A porcine model was selected to minimize the inter-individual variability and enable the collection of HVM image sequences under highly standardized conditions and of optimal visual quality. Moreover, septic shock represents an ideal setting for the incorporation of the variability in TVD, RBCv, and the presence of perfused and non-perfused capillaries. Fifty-three HVM image sequences that were recorded during the experiments were analyzed manually and using the algorithm: 25 in the septic shock group and 28 in the control group. All image sequences were of satisfactory quality.

**Fig. 4:**
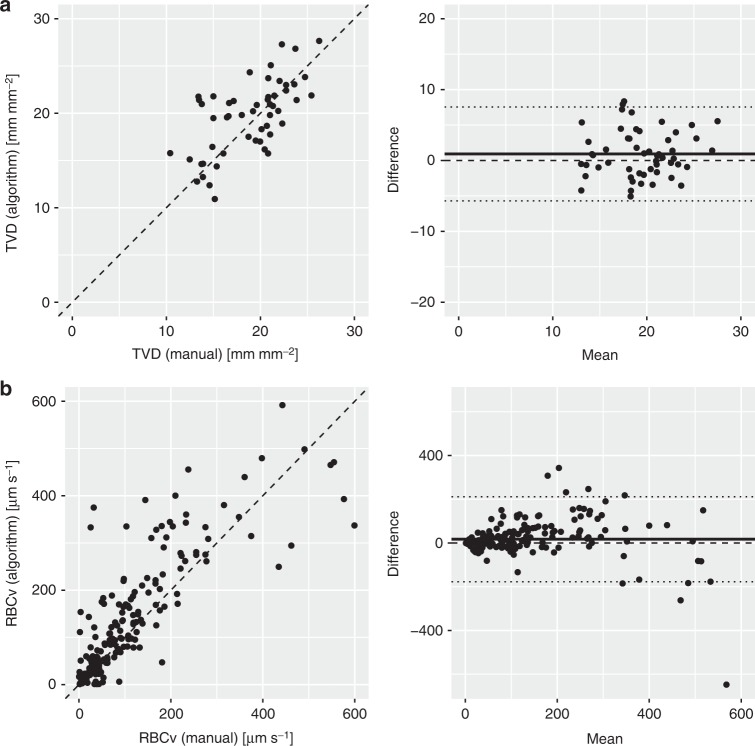
Good correlation was observed between manually measured and algorithm-based capillary TVD (**a**) and RBCv (**b**) in the septic shock model. TVD was compared by field of view in the septic shock and control groups (*n* = 53), and good correlation was observed (*r* = 0.7, *p* < 0.0001, bias 0.9 mm mm^−2^, level of agreement −5.7 to 7.5 mm mm^−2^, precision 3.3 mm mm^−2^, percentage error 6.7%). RBCv was compared by capillary in the septic shock group (*n* = 202), and good correlation was observed (*r* = 0.8, *p* < 0.0001, bias 17 µm s^−1^, level of agreement −117 to 212 µm s^−1^, precision 97 µm s^−1^). Dashed lines represent identity lines. In Bland–Altman analysis, solid line represents bias and dotted lines represent ±2*σ* levels of agreement. TVD total vessel density, RBCv red blood cell velocity

*Automated vessel recognition*: In 53 HVM image sequences, 2116 vessels were detected by the algorithm, 1922 of which were classified as capillaries. The overall capillary TVD was measured at 18.9 ± 3.9 manually and 19.9 ± 4.1 mm mm^−2^ using the algorithm (Table 2). Identification of the total lengths of all false-negative and false-positive detected capillaries by the algorithm compared to manual detection in each image sequence yielded a false-negative capillary density of 2.1 ± 1.5 (10%) and false-positive capillary density of 0.5 ± 0.4 mm mm^−2^ (2%). A good correlation between manually measured and algorithm-based capillary TVD across both groups and all timepoints was identified (*r* = 0.7, *p* < 0.0001). Bland–Altman analysis revealed a bias and a level of agreement of 0.9 (−5.7 to 7.5) mm mm^−2^, along with a precision and a percentage error of 3.3 mm mm^−2^ and 6.7%, respectively (Fig. 4a). In the experimental septic shock group, the TVD values that were detected manually and algorithmically were similar at baseline, after induction of septic shock and after resuscitation (Table 2).

**Table 2:** Development of microcirculatory parameters across induction of septic shock and resuscitation as reflected by manual analysis as well as using an advanced computer vision algorithm

		All individuals	Septic shock group							
			Baseline		Septic shock			Resuscitation		
		*n* = 17	*n* = 10		*n* = 10			*n* = 5		
		Mean ± SD	Mean ± SD	Mean ± SD	Estimate ± SE (CI)	*t* statistic	Mean ± SD	Estimate ± SE (CI)	*t* statistic	*p*
*Total vessel density (capillaries)*										
TVD (manual) [mm mm^−2^]		18.9 ± 3.9	19.7 ± 3.9	17.6 ± 5.2	−1.7 ± 1.1 (−3.7 to 0.2)	−1.53	16.9 ± 3.4	0.7 ± 1.0 (−0.9 to 2.4)	0.76	0.18
TVD (algorithm) [mm mm^−2^]		19.9 ± 4.1	22.3 ± 4.6	20.5 ± 4.6	−1.0 ± 1.5 (−3.4 to 1.5)	−0.66	20.9 ± 2.9	0.9 ± 1.3 (−1.2 to 3.1)	0.74	0.54
*Red blood cell flow velocity (capillaries)*										
MFI (manual) [1]		2.7 ± 0.5	2.9 ± 0.3	2.2 ± 0.5	−0.5 ± 0.2 (−0.8 to −0.2)	−2.62	2.2 ± 0.8	0.2 ± 0.2 (0.0–0.5)	1.55	0.01
RBCv (algorithm) [µm s^−1^]		232 ± 75	270 ± 78	165 ± 67	−73 ± 21 (−110 to −37)	−3.41	163 ± 75	44 ± 18 (41–57)	2.36	<0.001
*Functional capillary density*										
FCD (manual) [mm mm^−2^]		16.6 ± 4.1	18.0 ± 3.4	13.4 ± 3.9	−4.0 ± 1.3 (−6.2 to −1.8)	−3.11	12.3 ± 3.0	1.5 ± 1.1 (−0.5 to 3.4)	1.30	<0.01
PPV (manual) [1]		88 ± 10	92 ± 6	77 ± 11	−13 ± 4 (−19 to −7)	−3.55	74 ± 13	5 ± 3 (−1 to 10)	1.48	<0.01
FCD (algorithm) [mm mm^−2^]		16.7 ± 4.2	20.1 ± 4.0	14.8 ± 4.9	−3.9 ± 1.5 (−6.5 to −1.3)	−2.57	14.6 ± 2.5	2.1 ± 1.3 (−0.1 to 4.4)	1.60	<0.01
PPV (algorithm) [mm mm^−2^]		85 ± 13	91 ± 7	73 ± 18	−14 ± 5 (−22 to −6)	−2.98	71 ± 15	6 ± 4 (0–13)	1.56	<0.01

Timepoints were compared using a one-way mixed linear model analysis with timepoint as fixed effects and subject number as random effects. Estimates, standard error, confidence intervals, and *t* statistic are given for each timepoint. Full model *p* values were computed calculated using a likelihood ratio test. *TVD* total vessel density, *FCD* functional capillary density, *PPV* proportion of perfused vessels, *MFI* microvascular flow index, *RBCv* red blood cell velocity, *SD* standard deviation, *SE* standard error, *CI* confidence interval

*Automated space–time diagram generation and RBCv measurement*: The algorithm tracked 118 907 red blood cells across 5667 frames in 53 HVM image sequences. Overall, algorithm-based space–time analysis revealed an RBCv of 232±75 µm s^−1^ (Table 2). In the experimental septic shock group, a decrease of 19% in the manually detected MFI across the induction of shock was reflected in a decrease of 40% in the algorithmically measured RBCv (Table 2). MFI is a qualitative score that is specified in arbitrary units, in contrast to the values that are derived from space–time diagrams, which are quantitative (µm s^−1^). For the 202 randomly selected capillaries with manually generated space–time diagrams across all timepoints, a good correlation between manually determined and algorithm-based RBCv was identified (*r* = 0.8, *p* < 0.0001). Bland–Altman analysis revealed a bias and level of agreement of 17 (−117 to 212) µm s^−1^ and a precision of 97 µm s^−1^ (Fig. 4b). An increased variability in the RBCv difference was observed for higher mean values.

**Fig. 5:**
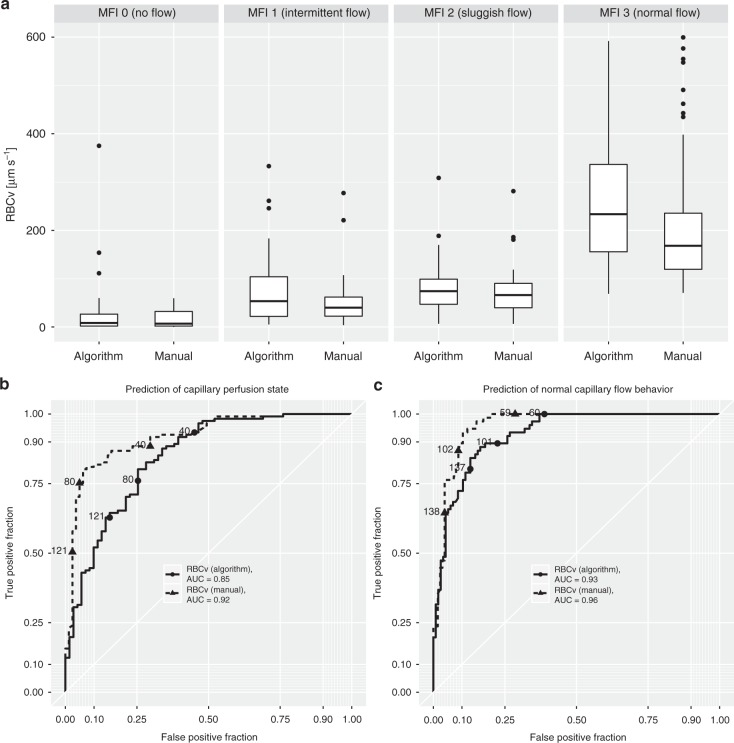
A gradual increase in algorithm-based and manually measured capillary RBCv with increasing MFI score across all timepoints in the septic shock model (**a**) is reflected in prediction of capillary perfusion state as defined by per-capillary MFI score ≥2 (**b**) and normal flow behavior as defined by per-capillary MFI score of 3 (**c**). RBCv was compared by capillary in the septic shock group (*n* = 202). Two-way mixed linear model analysis with MFI classification and analysis method as fixed effects and subject number as random effect demonstrated *p* < 0.0001 for the effect of MFI classification and *p* = 0.12 for analysis method. Boxplots represent median, interquartile range, and range. RBCv red blood cell velocity, MFI microcirculatory flow index, AUC area under the curve

**Fig. 6:**
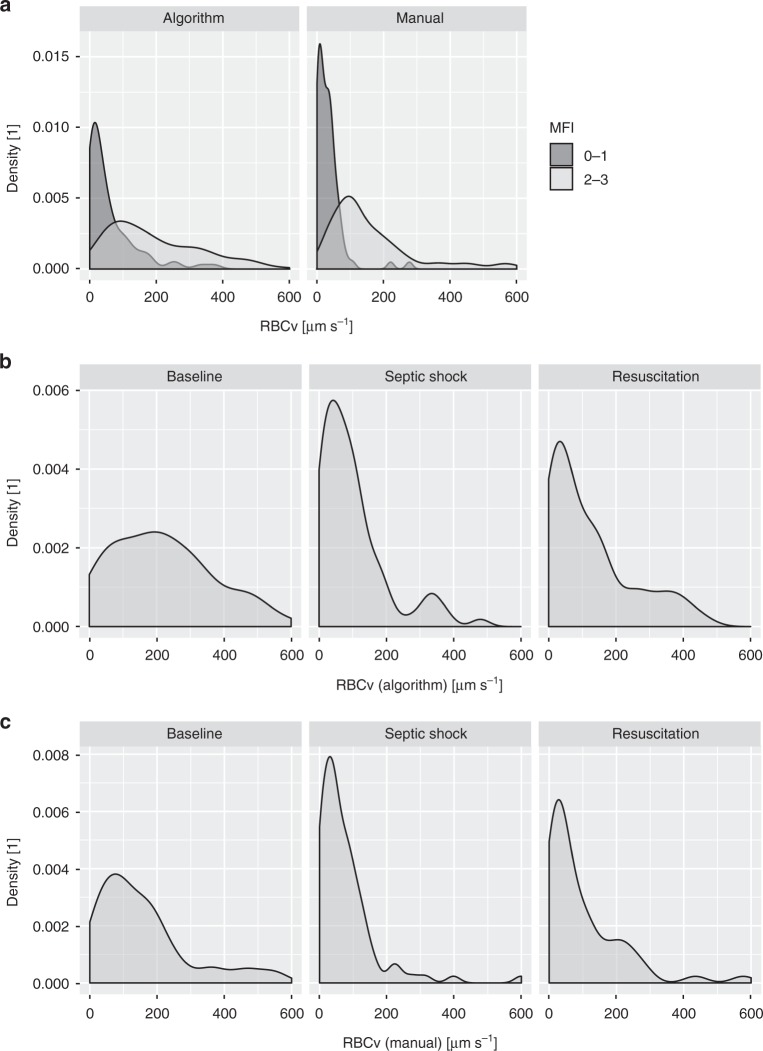
Density distribution of individual capillary RBCv as measured using algorithm-based and manually generated space–time diagrams demonstrate clear differentiation between perfused versus non-perfused capillaries across all timepoints in the septic shock model (**a**). Characteristic changes throughout induction of septic shock and resuscitation are revealed in RBCv density distributions measured using the algorithm (**b**) and manually (**c**). RBCv red blood cell velocity, MFI microcirculatory flow index

*Prediction of capillary perfusion state via space–time diagram-derived RBCv*: Categorized according to the four levels of the MFI score, a gradual increase in the capillary RBCv, as measured both manually and using the algorithm, was observed (*p* < 0.0001), whereas capillaries with MFI scores of 2 and 3 exhibited similar RBCv values (Fig. 5a). Receiver operating characteristics analysis revealed areas under the curve of 0.85 and 0.92 for the prediction of the capillary perfusion state as defined by MFI 0–1 and 2–3 for algorithm-based and manual analyses, respectively (Fig. 5b). Prediction of normal capillary flow behavior (MFI > 2) via the two methods of RBCv measurement yields areas under the curve of 0.93 and 0.96 (Fig. 5c). This finding is reflected in a clear differentiation of the density distributions of both manual and algorithm-based capillary RBCv values in capillaries with MFI 0–1 versus capillaries with MFI 2–3 (Fig. 6a).

Overall, PPV based on per-vessel subjective MFI and PPV based on algorithm-derived space–time diagrams correlated well (*r* = 0.8, *p* < 0.0001). Bland–Altman analysis revealed a bias and level of agreement of −3 (−20 to 14) % and a precision and percentage error of 9% and −3.3%, respectively (Supplementary Fig. 1A). Similar results are obtained for FCD that was derived using both methods (*r* = 0.7, *p* < 0.0001) (Supplementary Figure 1B), with Bland–Altman analysis revealing a bias and level of agreement of 0.2 (−6.0 to 6.3) mm mm^−2^ and a precision and percentage error of 3.1 mm mm^−2^ and 2.9%, respectively (Fig. 4b). In septic shock, decreases of 20% and 22% were observed in manually and algorithmically determined PPV, with a similar result for FCD (Table 2). Neither parameter recovered after resuscitation, according to both manual and algorithm-based measurements; in the original publication, the same result was obtained for this data set and confirmed with contrast-enhanced ultrasound measurements^25^. The density distribution of the capillary RBCv exhibits a left-shift of the density peak throughout the induction of septic shock, which is accompanied by a decrease in density of normal RBCv that spares a density peak in the high-RBCv range. Throughout resuscitation, the former changes were not reversed; however, the RBCv density was partially restored in the normal- and high- RBCv ranges (Fig. 6b, c).

### Application of the algorithm to clinical data

**Fig. 7:**
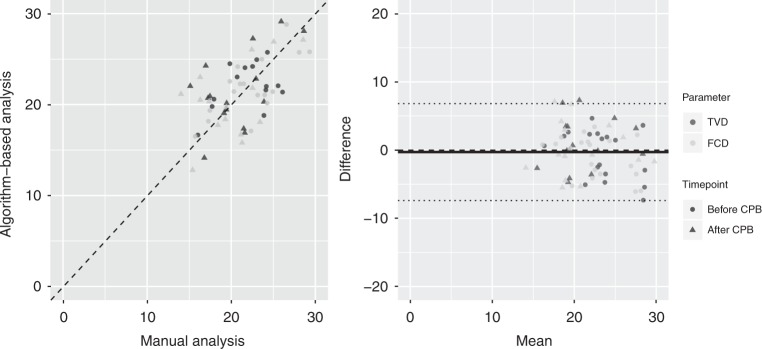
Good correlation was observed between manually measured and algorithm-based TVD and FCD throughout initiation of cardiopulmonary bypass. TVD and FCD were compared by field of view before and after initiation of cardiopulmonary bypass (*n* = 39). Dashed lines represent identity lines. Good correlation was observed for TVD (*r* = 0.7, *p* < 0.0001, bias 0.0 mm mm^−2^, level of agreement −7.3 to 7.3 mm mm^−2^, precision 3.6 mm mm^−2^, percentage error 1.9%) and FCD (*r* = 0.7, *p* < 0.0001, bias −0.6 mm mm^−2^, level of agreement −7.6 to 6.5 mm mm^−2^, precision 3.5 mm mm^−2^, percentage error −0.5 %). Dashed lines represent identity lines. In Bland–Altman analysis, solid line represents bias and dotted lines represent ±2*σ* levels of agreement for all data. TVD total vessel density, FCD functional capillary density

In recording and interpreting HVM image sequences that are obtained in clinical settings, challenges are encountered that are not present in a laboratory environment, such as compromised sublingual access and increased inter-individual variability. Thus, it is important for clinical applicability that an automated image analysis algorithm satisfy various robustness requirements. Cardiopulmonary bypass represents an ideal environment for studying parameters of diffusion and convection in HVM image sequences since the added extracorporeal circulation is expected to increase RBCv, while the hemodilution that is induced by rapid introduction of a large colloid priming volume into the cardiovascular system should decrease the density of capillaries that are perfused with red blood cells. Thirty-six HVM image sequences that were recorded during the experiments were analyzed manually and using the algorithm: 20 before and 16 after the initiation of cardiopulmonary bypass. The manual and algorithm-based analyses correlated well for TVD (*r* = 0.7, *p* < 0.0001, bias 0.0 mm mm^−2^, level of agreement −7.3 to 7.3 mm mm^−2^, precision 3.6 mm mm^−2^, and percentage error 1.9%) and FCD (*r* = 0.7, *p* < 0.0001, bias −0.6 mm mm^−2^, level of agreement −7.6 to 6.5 mm mm^−2^, precision 3.5 mm mm^−2^, and percentage error −0.5%) (Fig. 7). After the initiation of cardiopulmonary bypass, decreases in TVD and FCD were demonstrated via the manual and algorithm-based analyses (Table 3). The algorithm-based analysis also revealed an increase in RBCv, whereas qualitative grading of microvascular flow using the MFI score yielded “normal flow” both before and after the initiation of cardiopulmonary bypass (Table 3).

**Table 3:** Development of microcirculatory parameters across initiation of cardiopulmonary bypass as reflected by manual analysis as well as using an advanced computer vision algorithm

		Before CBP initiation		After CBP initiation		
		*n* = 11		*n* = 11		
		Mean ± SD	Mean ± SD	Estimate ± SE (CI)	*t* statistic	*p*
*Total vessel density (capillaries)*						
TVD (manual) [mm mm^−2^]		24.9 ± 1.3	20.9 ± 0.9	−4.0 ± 0.9 (−5.6 to −2.4)	−4.33	<0.001
TVD (algorithm) [mm mm^−2^]		23.5 ± 0.6	20.7 ± 1.0	−2.8 ± 0.8 (−3.1 to −1.4)	−3.55	<0.01
*Red blood cell flow velocity (capillaries)*						
MFI (manual) [1]		2.8 ± 0.1	2.8 ± 0.1	0.0 ± 0.1 (−0.2 to 0.2)	−0.28	0.78
RBCv (algorithm) [µm s^−1^]		330 ± 10	367 ± 10	38 ± 10 (35–56)	3.66	<0.01
*Functional capillary density*						
FCD (manual) [mm mm^−2^]		23.5 ± 1.2	19.2 ± 1.2	−4.3 ± 1.0 (−4.5 to −2.5)	−4.15	<0.01
PPV (manual) [1]		95 ± 1	91 ± 3	−3 ± 3 (−8 to 2)	−1.14	0.27
FCD (algorithm) [mm mm^−2^]		21.6 ± 0.7	19.2 ± 0.9	−2.3 ± 0.9 (−2.7 to −0.8)	−2.72	0.02
PPV (algorithm) [mm mm^−2^]		92 ± 1	93 ± 1	1 ± 1 (1–3)	1.16	0.26

Timepoints were compared using a one-way mixed linear model analysis with timepoint as fixed effects and subject number as random effect. Estimates, standard error, confidence intervals, and *t* statistic are given for each timepoint. Full model *p* values were computed calculated using a likelihood ratio test. *CPB* cardiopulmonary bypass, *TVD* total vessel density, *FCD* functional capillary density, *PPV* proportion of perfused vessels, *MFI* microvascular flow index, *RBCv* red blood cell velocity, *SD* standard deviation, *SE* standard error, *CI* confidence interval

### Time requirement for analysis

For the manual analysis of a total of 89 HVM image sequences, approximately 30 h of manual labor by an experienced human operator were required. In contrast, the algorithm-based analysis of 9013 frames in 89 HVM image sequences, including recognition and categorization of 5362 vessels and 175,650 RBC paths, was performed in 140 s on inexpensive and readily available consumer computing hardware. This corresponds to an analysis time of 0.388 s in real time per 1 s of microcirculatory monitoring.

## Discussion

In the present study, we propose a novel algorithm that is implemented in the MicroTools software package and employs advanced computer vision techniques for the automated analysis of HVM image sequences of the sublingual microcirculation, thereby meeting one of the expectations for future development of the 2018 International Consensus in the assessment of sublingual microcirculation in critically ill patients^9^. On the validation dataset, the proposed algorithm (I) enables automated measurement of TVD that is equivalent to manual analysis. Furthermore, via manual and algorithm-based analyses of space–time diagrams, (II) a consistent relationship between the subjective qualitative analysis of the capillary perfusion state and the absolute RBCv values that are based on space–time diagrams was demonstrated, thereby enabling the proposed algorithm to (III) reliably measure FCD and PPV as important functional parameters that are related to the physiological performance of the microcirculation. In this way, systematic automated space–time diagram analysis, as represented in the present study, enables for the first time the objective measurement of the absolute RBCv in HVM image sequences.

Early attempts at automated vessel recognition in HVM image sequences have struggled to model the inherent properties of HVM image sequences. Dobbe and co-workers^15^ used a simplified implementation of principal-curvature-based region detection, most likely to accommodate for the lower processing power that was available at the time. The method relies on several manual adjustments and, at best, may be used to assist in manual analysis. As a consequence, currently, the AVA 3.2 software is mainly used for its manual drawing capabilities. Bezemer and co-workers^16^ improved upon this approach by adding contrast-based detection of false-positive candidates via the vessel detection algorithm; however, they were not able to match the algorithm-based detection to manual image analysis via SDF imaging. Demir and co-workers^26^ used a combination of thresholding and the Euclidean distance transform to detect and automatically discriminate between perfused and non-perfused capillaries. Software packages building on further evolutions of such algorithms, as well as incorporating Frangi’s multiscale vessel enhancement filtering^27^, did not reproduce manual analysis to a satisfactory degree as has been shown for CCTools 1.7.x (Braedius Medical, Huizen, The Netherlands)^28^ and AVA 4 (Microvision BV, Amsterdam, The Netherlands)^29^. Thus, none of these methods have been widely adopted and manual analysis using AVA 3.2 has so far remained the gold standard for vessel recognition. Quantification of red blood cell flow velocity has constituted an even greater challenge. Measurement of RBCv based on manual generation of space–time diagrams and manual identification of a small proportion of the available red blood cell paths therein have been previously employed^13,24^. However, due to the practical inability to manually process the thousands of capillaries and hundreds of thousands of RBC paths that are contained in a typical research dataset and the associated introduction of bias, this method has not been widely used. Other approaches that are independent of space–time diagrams have been implemented. Bezemer and co-workers^16^ used temporal pixel intensity fluctuations that are consequent to red blood cell passage, which are quantified by the standard deviation of the intensities, to derive a relative parameter of red blood cell movement. CCtools 1.7.x has introduced an indicator of relative movement that is based on the intensity variation along the capillary centerlines; it is called the average perfused speed indicator. However, these were not quantitative measures, but provided relative indices^13,30^. These approaches were less dependent on the capability for reliable vessel recognition; however, they were hampered by their relative nature and their resulting in incomparability to other data. For these reasons, the scientific community has mostly relied on an entirely subjective, qualitative score of flow velocity—MFI. In contrast, our proposed algorithm realizes improved vessel recognition by introducing a combination of contrasting techniques that were demonstrated to improve preconditions for further analysis^26^ via the detection of discrete curvilinear structures with a high degree of independence from asymmetries in the background composition. In addition, the concept of discrete detection passes that are tailored to various different vessel structures, such as capillaries and venules, is introduced. This concept enables, for the first time, the systematic assessment of the absolute RBCv for all tracked red blood cell paths within every capillary and yields a meaningful representation of RBCv for the entire field of view by employing length-based weighting of RBCv in individual capillaries. Thus, the measure of RBCv derived for the field of view is rendered independent of the vessel segmentation and adheres to the physiological principle that longer capillaries contribute more to the capillary delivery of oxygen. Additionally, the proposed software package addresses concerns in current practice that originate from the use of outdated compression algorithms for HVM image sequences, such as the deterioration of the signal-to-noise ratio with every editing action in AVA 3.2 and the use of uncompressed raw data, which results in very large storage requirements, e.g., in the CCtools 1.7.x software. These concerns are addressed via the use of a newly developed, lossless compressed file format for the storage of raw data with embedded metadata that is based on the HFYU algorithm. Such files can be previewed on any standard-abiding video playback software.

As a measure of vessel recognition performance of the proposed algorithm, percentage error for algorithm-based TVD and false-negative and false-positive rates, compared to manual vessel recognition, of below 10% were found. Although in theory, a good correlation between manual and algorithm-based TVD may originate in separate structures whose total length would result in a similar TVD, the low false-negative and false-positive rates that are detected in our data indicate the detection of congruent vessel structures by both methods. Thus, measurements that are obtained using the proposed algorithm reflect the microcirculatory structure and physiology as accurately as manual analysis. Furthermore, the false-negative rate was found to be approximately fivefold higher than the false-positive rate, thereby increasing the robustness of the algorithm by reducing the random signal noise that is due to false recognition of the background structures as vessels. Previous data on the accuracy of manual analysis of HVM image sequences demonstrated an intra-observer variability of 9.6% and inter-observer variability of between 13% and 26%^31^. In contrast, the lower observed variability in algorithm-based TVD as compared to manual analysis in the present study is attributed to the elimination of intra-individual variability via the use of a clearly defined algorithm, and false-positive and false-negative rates well below the described inter-observer variability for manual analysis. The algorithm’s tendency after induction of septic shock to yield minimally larger values for TVD as compared to manual analysis may be due to inter-observer variability in manual analysis, differences in linking behavior of capillaries at intersections, or potential difficulties to correctly identify capillaries with abnormal red blood cell flow behavior by the manual operator.

Validation of the automated analysis of FCD and PPV was successfully achieved in three steps: First, clear differentiation of capillaries that were subjectively classified as perfused versus non-perfused according to space–time diagram-based measurements of RBCv was demonstrated. Second, demonstrating good correlation of the algorithm-based space–time diagram analysis, which was fundamentally enabled by automated recognition of capillaries as discussed above, with manual space–time diagram analysis was demonstrated. The increased variability in the RBCv difference between automated and manual that was observed at higher mean values may be due to the larger change in RBCv that results from a similar change in the slope angle of a red blood cell path in a space–time diagram in the high velocity range which is inherent to both algorithm-based and manual measurements. Third, good correlations among algorithm-based, space–time-diagram-derived and manual analysis, subjectively classified FCD and PPV and the finding of similar decreases in FCD and PPV throughout the induction of septic shock further support the relationship found between automated and manually derived FCD and PPV. It remains to be determined in future studies whether this relationship and the differentiation of subjective MFI score groups by space–time-diagram-derived RBCv are observed for examinations of the microcirculation that are obtained in other settings. Considering all four categories of the per capillary MFI score^20^, RBCv did not differentiate between MFI score categories 2 (intermittent flow) and 3 (sluggish flow). Thus, the receiver operating characteristics area under the curve for the identification of capillaries with subjectively classified normal flow (MFI 3) was greater than for the identification of capillaries considered perfused by the consensus definition (MFI 2–3)^9^. In the future, the algorithm may be extended to account for a continuum of changes in the red blood cell path velocity throughout a space–time diagram. The effect of this finding on the determination of capillary perfusion state by the algorithm is mitigated by the use of velocity density distributions of red blood cell paths within individual capillaries instead of a single RBCv cutoff value. The benefit of quantitative RBCv measurement in comparison to a subjective qualitative score is apparent across the initiation of cardiopulmonary bypass, where the added extracorporeal circulation was demonstrated to increase RBCv, whereas in the cardiopulmonary-stable patients who were entering elective surgery, the qualitative microvascular flow was normal at both timepoints. A potential source of noise in the algorithm-determined FCD, PPV, and RBCv parameters was infrequent as indicated by the small amount of false-positively recognized capillaries that did not yield meaningful red blood cell paths in the space–time diagram. The close correlation of these parameters between algorithm-based and manual measurement suggests that most of these red blood cell paths were correctly recognized by the algorithm as artifacts, and thus ignored.

We identify four fundamental advantages of automated microcirculatory image analysis: For the first time to our knowledge, the objective analysis of HVM image sequences can be separated from inter- and intra-operator variability during the analysis stage. Second, it introduces a quantitative measure of RBCv, which has the potential to replace previous qualitative and subjective parameters. Third, via systematic analysis of the position and movement of every single red blood cell that is within the field of view, a data-driven approach is introduced into HVM image sequence analysis, which enables the calculation of new parameters, such as the capillary blood volume (unit mm^3^ mm^−2^) and capillary blood flow (unit mm^4^ s^−1^ mm^−2^) within the field of view, that may more closely reflect the microcirculatory delivery of oxygen than previous parameters, as requested by the second consensus on the assessment of sublingual microcirculation in critically ill patients^9^, and potentially renders the obtained parameters more robust to noise. An expansion of the algorithm based on its ability to recognize capillaries and track red blood cells could further be utilized in the future to measure capillary hematocrit, thus enabling measurement of delivery of hemoglobin (unit mm^4^ s^−1^ mm^−2^). Using novel hardware to optically measure hemoglobin oxygen saturation would then allow for direct measurement of microcirculatory delivery of oxygen, a modality providing a physiological parameter of high clinical relevance. Finally, the technical prerequisites for clinical use of HVM are fulfilled by the combination of the demonstrated reliability of the automated analysis, complete independence from user intervention, and analysis speed approximately three times faster than real time using medium-range off-the-shelf computing hardware. For the first time to our knowledge, investigator-independent point-of-care analysis of the sublingual microcirculation may become feasible, resolving one of the main current concerns regarding microcirculation-targeted resuscitation^32–34^ aimed at titrating therapy that targets resolution of microcirculatory alterations associated with conditions of shock.

The software, as currently implemented, has two main limitations: First, the parameters of the algorithm may need to be adjusted for the analysis of HVM image sequences according to the species, tissue type, and type of camera used for recording, e.g., sidestream dark field or orthogonal polarization spectral imaging instead of the incident dark field image system for which the present software was developed here. This limitation is a direct consequence of the highly specialized nature of the algorithm, in contrast to a more generalized application of advanced computer vision that could adapt more flexibly to various types of input, and results in a highly specific relationship between the input and output and the promotion of consistent and highly reproducible results, as desired for scientific applications. Second, within the workflow for assessing microcirculatory parameters for research and clinical use, quality control of HVM image sequence data is of central importance. Automated quality assessment of image sequences must be implemented in the future at a point-of-care setting, using currently available criteria, such as Massey’s score^35^, as suggested by the current consensus^9^, or a novel system that could be adapted to the requirements of automated analysis. Such an approach to automatically assess the quality of images will require not only an automated identification of content, focus, and pressure artifacts but will also need to balance the analyzable image sequence length versus the stability and consecutively, field of view. Regarding the latter, for the validation of the current software high-resolution HVM image sequences recorded with the incident dark field technique were converted to AVA 3.2 format to enable direct comparison to the current gold standard. This step, imposed by the AVA 3.2 software, required a reduction of the field of view and resolution. In future studies, the proposed software will enable the use of the full field of view and resolution of any HVM image sequences for analysis. Further, even though the red blood cell velocities that may be measured using space–time diagrams and the currently available hardware are within the physiological range in most cases, it cannot be excluded that the actual velocity of some red blood cells exceeds this limit. This may be counteracted by increasing the frame rate in future HVM microscopes. Future studies could further explore differences in the sensitivity and precision of the methodology proposed in the current study by comparing the proposed algorithm to other techniques for measuring RBCv than space–time diagrams^36,37^, and validate the algorithm in large clinical datasets.

In conclusion, our proposed advanced computer vision algorithm has been demonstrated to reliably measure TVD, FCD, and PPV in HVM image sequences of the sublingual microcirculation with less than 10% error compared to manual analysis. In addition, we have demonstrated a consistent relationship between the subjective qualitative analysis of capillary perfusion state and the space–time-diagram-based absolute RBCv, thereby enabling the comparison of algorithm-based FCD and PPV with previous literature according to the current 2018 consensus. For the first time to our knowledge, it is possible to systematically quantify the displacement of red blood cells in HVM image sequences and analyze the velocity density distributions. Hence, our algorithm may pave the way towards real-time bedside analysis of the microcirculation and the development of novel parameters that more closely reflect the determinants of microcirculatory delivery of oxygen and discern patterns of microcirculatory heterogeneity that are induced by diseases such as sepsis. Use of the algorithm in conjunction with HVM in preclinical settings allows the application of information regarding microcirculatory alteration to be translationally applied to parallel clinical settings. In this way, assessment of microcirculatory function may complement point-of-care evaluation of disease severity and treatment response and ultimately be used as a target to counteract microcirculatory alterations that are known to be associated with adverse clinical outcome.

## Methods

### Input data, image enhancement, and development methodology

The proposed algorithm accepts microcirculatory image sequences of adequate quality as input (Fig. 1). HVM image sequence quality may be assessed according to the current consensus^9^ using Massey’s scoring system^35^, with a Massey score <10 considered adequate for analysis. Differences in image capture sensors and optical systems among discrete HVM devices (e.g., the Cytocam incident dark field HVM device, Braedius Medical, Huizen, The Netherlands; the Microscan sidestream dark field HVM device, Microvision BV, Amsterdam, The Netherlands; the Capiscope sidestream dark field HVM device, KK technology, Devon, United Kingdom, etc.), and routinely applied conversion procedures from one video/image format to another influence not only the resolution but also the pixel pitch, which determines the transformation of measured parameters to real-world units. Thus, the video/image file type is determined by the software based on the properties of the discrete HVM devices and the video/image format (Supplementary Table 1). Once the video/image sequence type has been identified, conversion factors that were determined using calibration images that were acquired with a calibration device are applied. This procedure may be repeated for upcoming combinations of HVM devices and video formats. Then, image sequence stabilization is performed using the calculation of optical flow for a sparse feature set via the iterative Lucas–Kanade method with pyramids^38^, thereby resulting in an affine transformation with translation, rotation, and scaling components that is applied to each frame in sequence after trajectory smoothing. Stabilization artifacts, such as moving black borders and reduced or variable frame size, are accounted for by cropping and automatically adjusting the of field of view, respectively. The software that implements the proposed algorithm was written in native C++ and adheres to the C++17 specification found in ISO/IEC 14882. The core code base consists of approximately 7000 lines of code, which are maintained using git 2.19.1, and links to OpenCV, which is an open-source advanced computer vision library^21^. The National Library of Medicine Insight Segmentation and Registration Toolkit^39^ is used to import microcirculatory image sequences that have been recorded using earlier software. Parallel processing is realized using the GNU parallel software package. The software, along with the OpenCV 3.4.3 and ITK 4.13.1 libraries, were compiled using CMake 3.10.2 and g++ 8.2.0 on Linux 4.18.0. All HVM image sequence analysis in the present study was performed on a system that was running Ubuntu Linux 18.04 and was equipped with 32 GB of random-access memory and a six-core Intel 8700K central processing unit with the capability of processing twelve concurrent threads.

### Vessel recognition

The mean gray scale values of the corresponding pixels across all frames contained in the stabilized image sequence are used to generate a mean image. Then, the mean image is used for multiple passes of vessel recognition: the first pass is used to detect vessel structures of diameter up to approximately 20–30 μm, which are classified as capillaries, and the second pass to detect vessel structures of diameter up to approximately 400 μm, which are classified as venules. In each pass, contrast-limited adaptive histogram equalization is applied to the mean image by deriving gray-level assignment at each given position via bi-linear interpolation of the gray-value distributions in the surrounding contextual regions according to the following formula:

$$s \prime = \left( {1 - y} \right)\;\left( {\left( {1 - x} \right)\;g_A\left( s \right) + x\;g_B\left( s \right)} \right) + y\left( {\left( {1 - x} \right)\;g_C\left( s \right) + xg_D\left( s \right)} \right),$$

where *s* is the grayscale value of the pixel in question, *gA–D* are the grayscale values at the corners of the boundary rectangle of the surrounding contextual region, and *x* and *y* are normalized distances with respect to point *A*. Additionally, the slope of the brightness histogram that is associated with the gray-level assignment is limited to prevent the amplification of noise. The procedure is described in more detail elsewhere^40^. Then, a modified principal-curvature-based region detection algorithm for unbiased detection of curvilinear structures, which was initially described by Steger^41^, is then used for vessel detection. The algorithm combines convolution of the contrast-enhanced time-based mean image with first- and second-derivative Gaussian kernels and an orientation-based linking algorithm. The second-derivative Gaussian kernel is expressed by the following formula:

$$g \prime\prime_\sigma \left( x \right) = x^2 - \sigma ^2/\left( {\left( {2\pi } \right)^{ - 1/2}\;\sigma ^5} \right)\;{\mathrm{e}}^{ - x^2/\left( {2\sigma ^2} \right)},$$

where *σ* represents the kernel’s standard deviation, which mainly determines the properties of the linear structures that are recognized by the full algorithm^41^. A linking algorithm then analyzes the distance between the respective convolution line locations and the angle difference of the two points (parameters *h* and *l*, see Supplementary Table 2). Finally, this algorithm identifies the discrete vessel structures, which are described by a centerline and for each point therein, the direction of the normal vector and the vessel diameter. The algorithm’s effectiveness for vessel recognition in HVM image sequences is based upon two principles: First, it targets the detection of curvilinear structures such as capillaries and venules. Second, the use of model-line-profile scale-space behavior analysis subsequent to kernel convolution effectively counteracts differences in contrast levels on both sides of the vessel structures that arise from spatial variability of tissue properties, lighting, or superimposition with other structures. The parameters that are used in the vessel detection algorithm are listed in Supplementary Table 2. Then, the results of each vessel detection pass are then superimposed onto one another while eliminating overlapping structures, thereby yielding a final vessel map that contains a centerline and, for each line point, the vessel diameter.

### Space–time diagram-based RBCv estimation

Before a space–time diagram is generated for each capillary, differences in brightness and contrast in between frames of the image sequence are eliminated by applying of brightness histogram equalization, followed by Gaussian smoothing, to each individual frame. Then, columns of pixels are read out along the centerline of each capillary from each frame and appended horizontally. Prior to further processing, brightness histogram equalization is applied again to each space–time diagram. In this way, space–time diagrams are generated for all vessels in the field of view, which are subsequently used to measure the motion of individual red blood cells within the field of view^24^. The same principal-curvature-based region detection algorithm is applied to each space–time diagram as for vessel recognition. The resulting centerlines represent the paths of individual red blood cells along the space–time axes, with the mean of its first derivative with respect to time representing RBCv for an individual red blood cell path. Paths are classified as artifacts and discarded based on three criteria: they are discarded if their length is below a minimum path length, if the ratio of length of a straight line between the start- and endpoints of the path and the actual path length—which is called the curvature index—is below a specified cutoff, or if RBCv exceeds the maximum detectable RBCv, which depends on the length of the vessel (*l*; μm) and the frame rate of the video/image sequence (*f*; s^−1^) and is expressed as

$$v_{\mathrm{{max}}} = 3^{ - 1}\;l\;f\;\left[ {\upmu {\mathrm{m}}\;{\mathrm{{s}}}^{ - 1}} \right]$$

as previously described^15^. The remaining red blood cell paths are classified as low flow or normal flow based on a species-specific critical RBCv cutoff value (low_flow_cutoff, see Supplementary Table 2). Then, the vessel RBCv is derived as the mean RBCv of all red blood cell paths within one space–time diagram, whereas the same principle is applied to capillaries and venules, as was demonstrated previously^13^. Vessels are classified as perfused if the proportion of normal-flow red blood cell paths in a per-vessel density distribution lies above 2*σ* of a fitted normal distribution (>95%), whereas density distribution-based vessel perfusion classification is used to increase the robustness to red blood cell path artifacts as compared to the simple application of the RBCv cutoff to the vessel RBCv (see Fig. 1).

*Elimination of confounders in space–time diagram analysis*: Space–time diagrams are susceptible to several confounders. First, in manually assisted generation of space–time diagrams small deviations in the vessel centerline are inevitably introduced during manual drawing. Such deviations can introduce noise into space–time diagrams that are generated from the pixels along the vessel centerline and can be minimized using automated vessel recognition. Second, because they rely on the composition of the pixels that are extracted from all consecutive frames, differences in brightness between frames (flickering) may introduce marked vertical stripes characteristics into space–time diagrams that are produced without further image processing, e.g., by the AVA 3.2 software (Fig. 3a–d, top- and bottom-left images)^17^. Flickering may originate from technical limitations of recording equipment, such as an undesired rapid change in exposure; non-stationary illumination brightness; or by changes in the blood flow in the background. Frame-by-frame optimization, as performed by the proposed algorithm, effectively eliminates these artifacts, thereby avoiding contamination of detected red blood cell paths by these artifacts and enabling optimal red blood cell transit time recognition (Fig. 3a–d, top- and bottom-right images). Third, above the upper limit of detection of RBCv, which is determined by the vessel length and the frame rate, it is difficult to distinguish non-moving red blood cell paths from artifacts since both may present themselves as near-horizontal lines. In this case, non-moving red blood cell paths are classified as such if the red blood cell paths below a cutoff velocity make up more than 2*σ* of a fitted normal distribution (>95%) of all detected red blood cell path velocities within the same vessel; otherwise, they are classified as artifacts. If this cutoff has been reached in a vessel, the vessel is considered non-perfused regardless of the velocity of a minority of red blood cell paths that are contained therein. In this way, the “barcode sign” space–time diagram configuration of a capillary with no flow (Fig. 3d) is reliably recognized.

### Output parameters and formats

Parameters for all detected structures are written to three human- and machine-readable files that contain the results for each image sequence, vessel and red blood cell path, enabling further analysis on a per-field of view, per-vessel, or per-red blood cell basis. The calculated parameters are listed in Table 1. The stabilized video/image sequence file is written to file using the HFYU lossless codec; visualizations of detected vessels, space–time diagrams, and individual RBC paths may be written to image files using the PNG lossless codec (ISO/IEC 15948).

### Validation of the algorithm

*Validation of vessel recognition*: In a porcine model of septic shock, 17 female pigs (crossbred Landrace × Yorkshire, 3–4 months old) were divided into a septic shock group (*n* = 10) and a control group (*n* = 7). In the former group, sublingual HVM image sequences were recorded with a Cytocam HVM device before and after induction of septic shock (mean arterial pressure <60 mmHg or serum lactate >2.0 mmol l^−1^) using intravenous infusion of lipopolysaccharide (LPS; *Escherichia coli* LPS 026:B6; Difco Laboratories, Detroit, MI) at 2 μg kg^−1^ h^−1^ and 1 h after normalization of the mean arterial pressure via fluid resuscitation. Data obtained from the experiments have been described elsewhere^25^. Septic shock was defined as a mean arterial pressure <60 mmHg or serum lactate >2.0 mmol l^−1^. In the control group, the experiment was performed without the administration of LPS. The study was conducted with permission from the local animal experimental committee (EMC3379 142-14-01) and in strict accordance with the National Guidelines for Animal Care and Handling. For gold-standard measurements of TVD, FCD, and PPV, all HVM image sequences were manually analyzed using AVA 3.2 software by a single, experienced operator in a blinded manner^25^. Then, these measurements were compared to the results of a fully unattended, algorithm-based analysis. In an additional step, all vessels that were detected by the algorithm were electronically superimposed onto the image sequences, thereby enabling manual identification of all false-negative and false-positive vessel segments according to the operator’s judgment, as in the manual analysis using the AVA 3.2 software. The false-negative and false-positive TVD values were calculated by normalizing the total length of all false-negative and false-positive capillaries by the field of view. The dataset including the sublingual microcirculation analyzed using the AVA 3.2 software has been previously published elsewhere^25^.

*Space–time diagram-based RBCv and capillary perfusion*: The calculations of FCD and PPV according to the current consensus are based on capillary perfusion state classification according to the per-capillary MFI^9,42^. To examine the relationship between this subjective semi-quantitative analysis and the space–time-diagram-derived RBCv, between 5 and 20 capillaries were randomly selected from HVM image sequences that were obtained in the septic shock group. The capillaries’ centerlines were manually drawn in the AVA 3.2 software to match the lengths and positions of capillaries that were identified by the algorithm. These capillaries were manually graded using the MFI score (0: no flow, 1: intermittent flow, 2: sluggish flow, and 3: normal flow)^18^ and classified as non-perfused (MFI score 0–1) or perfused (MFI score 2–3) according to the current guidelines^9,42^. Then, space–time diagrams were generated using AVA 3.2 and three to five red blood cell paths were manually identified in each space–time diagram, whereas the manually measured capillary RBCv was calculated as the mean slope of all RBC paths. An analysis of 202 manually generated space–time diagrams that were obtained in this way was correlated to a fully unattended, algorithm-based space–time diagram analysis. The predictive values of the manual and algorithm-based space–time-diagram-derived RBCv values for subjective MFI scoring in the same capillaries were evaluated. Representative examples of automatically generated space–time diagrams and red blood cell path detection are presented for capillaries with normal flow (Fig. 3a), “hyperdynamic” flow (Fig. 3b), intermittent flow (Fig. 3c), and no flow (Fig. 3d).

*Clinical dataset*: Sublingual HVM image sequences were recorded at three differing sublingual locations in 11 patients who were undergoing elective coronary artery bypass surgery (38% male, age 59 ± 11 years, weight 78.6 ± 3 kg, and height 1.7 ± 0.1 m) both after induction of anesthesia and again after initiation of cardiopulmonary bypass. On the day of surgery, anesthesia was induced via bolus application of midazolam (50 μg kg^−1^ i.v.), fentanyl (30 μg kg^−1^ i.v.), and pancuronium (2 mg kg^−1^ i.v.). Then, cardiopulmonary bypass, primed with a colloid solution (HES 130/0.4; Fresenius Kabi, Bad Homburg, Germany), was initiated. HVM image sequences were graded in terms of quality using Massey’s scoring system^35^ and selected for analysis if the Massey score <10. The study was conducted with permission from the institutional Ethics Board of Acibadem University (ATADEK 2014/723) and after obtaining informed consent preoperatively from each patient.

### Statistics and reproducibility

TVD, FCD, and the RBCv values of individual capillaries as measured manually and with the algorithm were compared using a linear correlation that employed Pearson’s product-moment correlation coefficient alongside Bland–Altman analysis^43^ with percentage error analysis^44^. In the septic shock group, all manually measured and algorithm-derived microcirculatory parameters were compared at the baseline, septic shock, and resuscitation timepoints, respectively, before and after the initiation of cardiopulmonary bypass, using linear mixed-effect model analysis^45^. The effects in question were entered as fixed effects, and intercepts for subjects and per-subject random slopes representing the effect on the dependent variables were entered as random effects. *P* values were calculated using a likelihood ratio test of the full model with the effect in question against a “null model” that lacks the effect in question^46^. *P* values for individual fixed effects were obtained via the Satterthwaite approximation^47^. The predictive value of the perfusion state of capillaries according to MFI classification by the space–time-diagram-generated RBCv was examined using receiver operating characteristics analysis and calculation of the area under the curve. A two-sided *p* < 0.05 was considered statistically significant. Reproducibility was ensured by providing the dataset that supports the conclusions of this article in the Zenodo repository^48^, and utilizing a fully scripted data management pathway within the R environment for statistical computing, version 3.4.1. Receiver operating characteristics analysis was performed using the R library plotROC version 2.2.1. Graphical output was generated using the R library ggplot2, version 2.2.1. Values are specified as the mean ± standard deviation (SD).

### Ethics approval

Study of the porcine model of septic shock was conducted with permission from the local animal experimental committee (EMC3379 142-14-01) and in strict accordance with the National Guidelines for Animal Care and Handling. Patient data during cardiac surgery were recorded with approval of the institutional Ethics Board of Acibadem University (ATADEK 2014/723).

### Consent to participate and consent for publication

Informed consent from each patient was obtained preoperatively.

### Reporting Summary

Further information on research design is available in the Nature Research Reporting Summary linked to this article.

## Supplementary information

Supplementary Information

Reporting Summary

## Data Availability

The dataset that supports the conclusions of this article, encompassing microcirculatory parameters on an individual subject level that were used for validation of the algorithm in the experimental and clinical settings, is available in the Zenodo repository^48^. All other data are available from the corresponding author on reasonable request.

## Code availability

MicroTools source code is available via the github software version control repository. The repository has been uniquely identified and archived in the Zenodo repository^49^. The code is published under the GPLv3 licence. For further availability of the software in the future, see https://microcirculationacademy.org.
