# Supplementary material for: MicroTools enables automated quantification of capillary density and red blood cell velocity in handheld vital microscopy
Source: Commun Biol. 2019 Jun 19;2:217. doi: 10.1038/s42003-019-0473-8 (PMC6584696; doi:10.1038/s42003-019-0473-8)
Supplement: Supplementary file 1 — Supplementary Information [file 42003_2019_473_MOESM1_ESM.pdf]

Supplementary Tables

HVM device	*Video format*	*Description*	*Approximate field of view* *	*Approximate pixel pitch* *
Cytocam IDF	avi, ≤ 716 x 572 px	CCtools AVA 3.x export	0.5898 mm^2	1.2 $\mu m\ px^{-1}$
Cytocam IDF	mha, ≤ 2208 x 1648 px	CCtools 1.7.x native recording format	1.7830 mm^2	0.7 $\mu m\ px^{-1}$
Microscan SDF	avi, ≤ 716 x 572 px	AVA 3.x native recording format	0.5898 mm^2	1.2 $\mu m\ px^{-1}$

*Supplementary Table 1. Combinations of HVM recording devices and video formats. "≤" denotes the possibility of frame downsizing during stabilization procedures preceding analysis. *field of view and pixel pitch were determined for each combination of individual HVM recording device and video format using a formal calibration procedure, approximate mean values are given here for reference. IDF, incident dark field; SDF, sidestream dark field.*

Symbol	*Description*		*Parameter values*<br>*Cytocam IDF, porcine sublingual microcirculation, AVA 3.x avi*
Vessel recognition			
clahe_clip	Contrast enhancement limit for contrast limited adaptive histogram equalization.	The number of segmentation passes is given by the number of parameters listed in the configuration file.	2, 0
clahe_grid [px]	Grid size for contrast limited adaptive histogram equalization.		32, 0
σ [px]	Standard deviation of the the Gaussian kernel used for principal curvature-based region detection[37].		3, 9
h [px]	Upper threshold for the linking algorithm, where new lines are created as long as the starting point has a higher second directional derivative[37].		1.1, 0.1
l [px]	Lower threshold for the linking algorithm, where points are added to the current line as long as they have a higher second directional derivative[37].		0, 0
min_length [px]	Threshold for a high-pass filter for vessel length implemented downstream of the detection and linking algorithms.		40, 120
capillary_cutoff [µm]	Low-pass cutoff value for mean vessel diameter used for vessel classification as a capillary.		20
Space-time diagram analysis			
smooth_σ [px]	Standard deviation of the Gaussian kernel used for Gaussian smoothing.		1
σ [px]	Standard deviation of the Gaussian kernel used for principal curvature-based region detection[37].		1.5
h [px]	Upper threshold for the linking algorithm, where new lines are created if the starting point has a higher second directional derivative[37].		1.1
l [px]	Lower threshold for the linking algorithm, where points are added to the current line if they have a higher second directional derivative[37].		0
min_length [px]	High-pass cutoff value for artefact detection: Threshold for a high-pass filter for vessel length implemented downstream of the detection and linking algorithms.		20
curvature index_cutoff [1]	High-pass cutoff value for artefact detection: proportion of length of straight line between start and endpoint of ridge AND actual ridge length.		0.02
low_flow_cutoff [$\mu m\ s^{-1}$]	Low-pass cutoff value for individual RBC path RBCv for RBC path classification of no flow/low flow versus normal flow.		10
perfused_cutoff [1]	High-pass cutoff value for the proportion of no flow / low flow RBC paths in a per-vessel frequency distribution used for vessel classification as perfused.		0.1

*Supplementary Table 2. Set of parameters for vessel detection and space-time diagram analysis using the proposed advanced computer vision algorithm. * depending on validation of combinations of tissue type, HVM recording devices and video formats.*

Supplementary Figures

Supplementary Figure 1. Good correlation was observed between manually measured and algorithm-based FCD (A) and PPV (B) in the septic shock model.

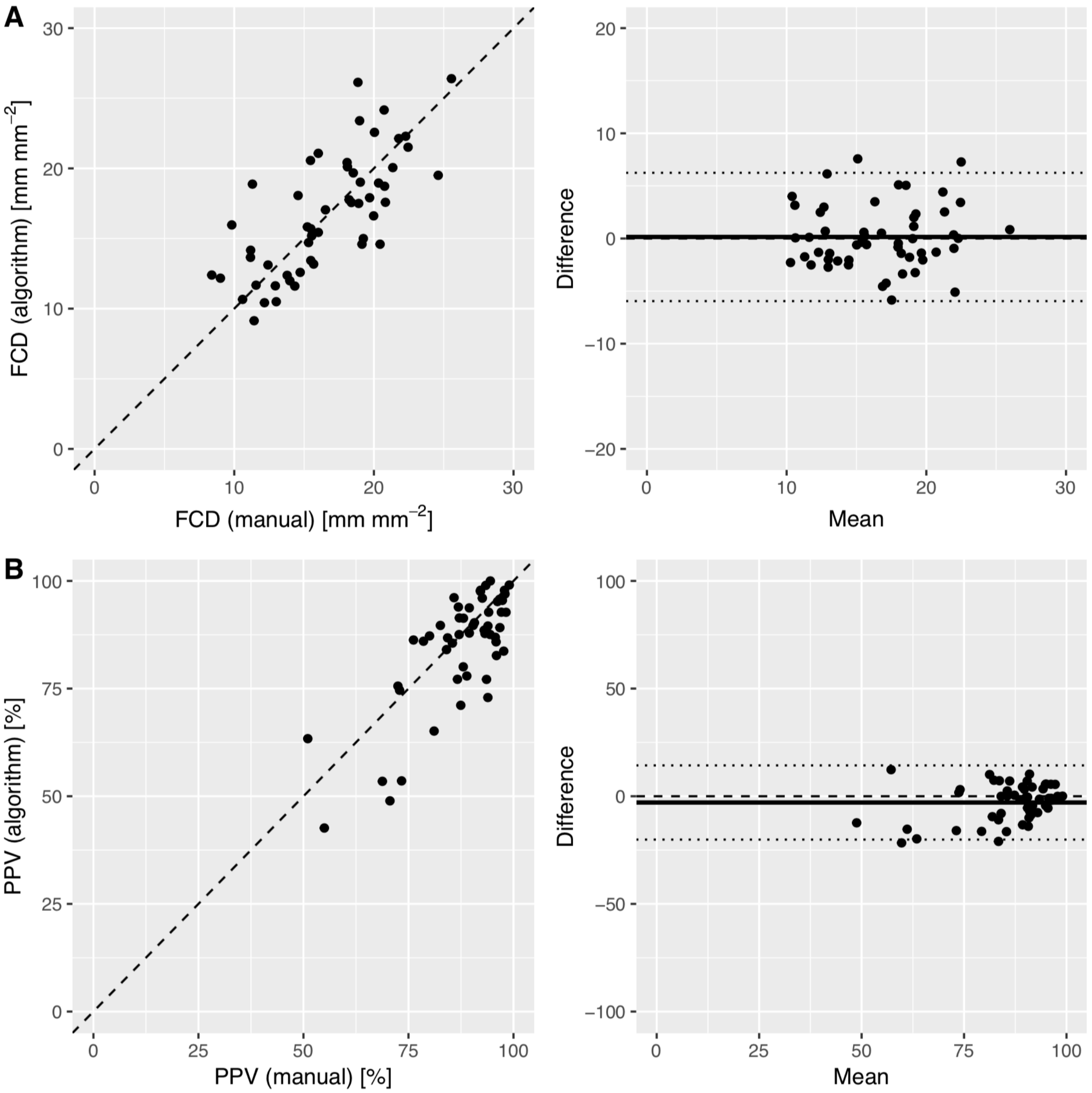

FCD and PPV were compared by field of view in the septic shock and control groups (n = 53). Good correlation was observed for FCD (r = 0.7, p < 0.0001, bias 0.2 mm mm^{-2}, level of agreement -6.0–6.3 mm mm^{-2}, precision 3.1 mm mm^{-2}, percentage error 2.9%) and PPV (r = 0.8, p < 0.0001, bias -3%, level of agreement -20–14%, precision 9%, percentage error -3.3%). Dashed lines represent identity lines. In Bland-Altman analysis, solid line represents bias and dotted lines represent ± 2 σ levels of agreement. FCD, functional capillary density; PPV, proportion of perfused vessels.
