# Supplementary material for: MicroTools enables automated quantification of capillary density and red blood cell velocity in handheld vital microscopy
Source: Commun Biol. 2019 Jun 19;2:217. doi: 10.1038/s42003-019-0473-8 (PMC6584696; doi:10.1038/s42003-019-0473-8)
Supplement: Supplementary file 2 — Reporting Summary [file 42003_2019_473_MOESM2_ESM.pdf]

nature research

Corresponding author(s): Matthias Hilty

Last updated by author(s): May 2, 2019

Reporting Summary

Nature Research wishes to improve the reproducibility of the work that we publish. This form provides structure for consistency and transparency in reporting. For further information on Nature Research policies, see Authors & Referees and the Editorial Policy Checklist.

Statistics

For all statistical analyses, confirm that the following items are present in the figure legend, table legend, main text, or Methods section.

n/a	Confirmed	
☐	☒	The exact sample size (n) for each experimental group/condition, given as a discrete number and unit of measurement
☐	☒	A statement on whether measurements were taken from distinct samples or whether the same sample was measured repeatedly
☐	☒	The statistical test(s) used AND whether they are one- or two-sided<br>*Only common tests should be described solely by name; describe more complex techniques in the Methods section.*
☐	☒	A description of all covariates tested
☐	☒	A description of any assumptions or corrections, such as tests of normality and adjustment for multiple comparisons
☐	☒	A full description of the statistical parameters including central tendency (e.g. means) or other basic estimates (e.g. regression coefficient) AND variation (e.g. standard deviation) or associated estimates of uncertainty (e.g. confidence intervals)
☐	☒	For null hypothesis testing, the test statistic (e.g. F, t, r) with confidence intervals, effect sizes, degrees of freedom and P value noted<br>*Give P values as exact values whenever suitable.*
☒	☐	For Bayesian analysis, information on the choice of priors and Markov chain Monte Carlo settings
☒	☐	For hierarchical and complex designs, identification of the appropriate level for tests and full reporting of outcomes
☐	☒	Estimates of effect sizes (e.g. Cohen's d, Pearson's r), indicating how they were calculated

Our web collection on statistics for biologists contains articles on many of the points above.

Software and code

Policy information about availability of computer code

Data collection	Handheld vital microscopy image sequences were recorded using the accompanying software to the Cytocam device: CCTools version 1.7.12 (Braedius Medical, Huizen, The Netherlands).
Data analysis	For data analysis, the algorithm implemented in the MicroTools software is described in the manuscript. MicroTools source code is available via the github software version control repository. The repository has been uniquely identified and archived in the Zenodo repository (DOI: 10.5281/zenodo.2608759). The code is published under the GPLv3 licence. Statistical analysis was peromed using a fully reproducible data management pathway within the R environment for statistical computing, version 3.4.1.

For manuscripts utilizing custom algorithms or software that are central to the research but not yet described in published literature, software must be made available to editors/reviewers. We strongly encourage code deposition in a community repository (e.g. GitHub). See the Nature Research guidelines for submitting code & software for further information.

Data

Policy information about availability of data

All manuscripts must include a data availability statement. This statement should provide the following information, where applicable:
- Accession codes, unique identifiers, or web links for publicly available datasets
- A list of figures that have associated raw data
- A description of any restrictions on data availability

The dataset that supports the conclusions of this article is available in the Zenodo repository: Hilty MP, et al. Zenodo data repos, DOI: 10.5281/zenodo.1489063.

Field-specific reporting

Please select the one below that is the best fit for your research. If you are not sure, read the appropriate sections before making your selection.

☒ Life sciences ☐ Behavioural & social sciences ☐ Ecological, evolutionary & environmental sciences

For a reference copy of the document with all sections, see nature.com/documents/nr-reporting-summary-flat.pdf

Life sciences study design

All studies must disclose on these points even when the disclosure is negative.

Sample size	The animal and human sublingual microcirculatory recordings used to validate the algorithm and software described in the present article were taken from two previous studies with separately obtained protocols and ethical approval, which are referenced in the present article. Both studies were suffitiently powered to reach their conclusions. The former has already been published and referenced in the present article, the latter is submitted for publication. All handheld vital microscopy image sequences from both studies were used in the present article for validation of the algorithm and software.
Data exclusions	The complete handheld vital microscopy image sequence dataset as used in the two underlying studies were used in the present article.
Replication	The output of the Microtools software, described in the present article, is reproducible as it follows the described algorithm.
Randomization	In the animal study, animal subjects were randomized into the control and sepsis groups. The human study was observational, thus no randomization was necessary.
Blinding	Investigators performing manual analysis of the handheld vital microscopy image sequences were blinded by randomly assigned file names.

Reporting for specific materials, systems and methods

We require information from authors about some types of materials, experimental systems and methods used in many studies. Here, indicate whether each material, system or method listed is relevant to your study. If you are not sure if a list item applies to your research, read the appropriate section before selecting a response.

Materials & experimental systems

n/a	Involved in the study	
☒	☐	Antibodies
☒	☐	Eukaryotic cell lines
☒	☐	Palaeontology
☐	☒	Animals and other organisms
☐	☒	Human research participants
☒	☐	Clinical data

Methods

n/a	Involved in the study	
☒	☐	ChIP-seq
☒	☐	Flow cytometry
☒	☐	MRI-based neuroimaging

Animals and other organisms

Policy information about studies involving animals; ARRIVE guidelines recommended for reporting animal research

Laboratory animals	The separate animal study from which the handheld vital microscopy image sequences were used in the present article used 17 female pigs, crossbred Landrace x Yorkshire, or 3-4 months of age.
Wild animals	The study did not involve wild animals.
Field-collected samples	The study did not involve samples collected from the field.
Ethics oversight	The study was approved by and guided by the Erasmus Medical Center animal experimental committee, Rotterdam, The Netherlands, protocol number EMC3379 142-14-01.

Note that full information on the approval of the study protocol must also be provided in the manuscript.

Human research participants

Policy information about studies involving human research participants

Population characteristics	The separate observational human study from which the handheld vital microscopy image sequences were used in the present article was performed in cardiopulmonary stable patients with diagnosed coronary artery disease, undergoing elective coronary

	artery bypass surgery. They had a (mean+/-SD) age of 59+/-11 years, weight of 78.6+/-3 kg, and height of 1.7+/-0.1 m and were of 38% male sex.
Recruitment	11 consecutive patients undergoing elective coronary artery bypass surgery at the Abicadem University, Istanbul, Turkey, were recruited after obtaining informed consent.
Ethics oversight	The study was approved by the Abicadem University institutional ethics board, Istanbul, Turkey, protocol number ATADEK 2014/723.

Note that full information on the approval of the study protocol must also be provided in the manuscript.
